# Risk and protective factors for mental health problems in preschool-aged children: cross-sectional results of the BELLA preschool study

**DOI:** 10.1186/s13034-017-0149-4

**Published:** 2017-03-08

**Authors:** Olga Wlodarczyk, Silke Pawils, Franka Metzner, Levente Kriston, Fionna Klasen, Ulrike Ravens-Sieberer, the BELLA Study Group, Ulrike Ravens-Sieberer, Fionna Klasen Claus Barkmann, Monika Bullinger, Manfred Döpfner, Beate Herpertz-Dahlmann, Heike Hölling, Franz Petermann, Aribert Rothenberger, Sylvia Schneiderr, Michael Schulte-Markwort, Robert Schlack, Frank Verhulst, Hans-Ulrich Wittchen

**Affiliations:** 1 Institute and Outpatients Clinic of Medical Psychology, Centre for Psychosocial Medicine, University Medical Centre Hamburg-Eppendorf, Martinistrasse 52 (Building W26), 20246 Hamburg, Germany; 2 Department of Child and Adolescent Psychiatry, Psychotherapy and Psychosomatics, Research Division “Child Public Health”, Centre for Psychosocial Medicine, University Medical Centre Hamburg-Eppendorf, Martinistrasse 52 (Building W26), 20246 Hamburg, Germany

**Keywords:** Mental health, Preschool, Cross-sectional studies, Risk factor, Protective factor

## Abstract

**Background:**

Mental health problems (MHPs) in preschoolers are precursors of mental disorders which have shown to be associated with suffering, functional impairment, exposure to stigma and discrimination, as well as enhanced risk of premature death. A better understanding of factors associated with MHPs in preschoolers can facilitate early identification of children at risk and inform prevention programs. This cross-sectional study investigated the association of risk and protective factors with MHPs within a German representative community sample.

**Methods:**

MHPs were assessed in a sample of 391 preschoolers aged 3–6 years using the Strength and Difficulties Questionnaire (SDQ). The effects of parental MHPs, children’s temperament, parental socioeconomic status (SES), social support and perceived self-competence on MHPs were assessed using bivariate and multivariate logistic regression analyses that controlled for sociodemographic characteristics.

**Results:**

Overall, 18.2% of preschoolers were classified as ‘borderline or abnormal’ on the total difficulties score of the SDQ. Bivariate analyses showed that parental MHPs, children’s difficult temperament, and parental low SES increased the likelihood, whereas high perceived parental competence decreased the likelihood of preschool MHPs. In the multivariate analyses, only difficult child temperament remained significantly associated with preschool MHPs when other variables were controlled.

**Conclusions:**

The results underline the importance of children’s difficult temperamental characteristics as a risk factor for mental health in preschoolers and suggest that these may also be an appropriate target for prevention of preschool MHPs. More research on specific aspects of preschool children’s temperament, the socioeconomic environment and longitudinal studies on the effects of these in the development of preschool MHPs is needed.

**Electronic supplementary material:**

The online version of this article (doi:10.1186/s13034-017-0149-4) contains supplementary material, which is available to authorized users.

## Background

Mental disorders are amongst the leading causes of disability and economic costs to public health worldwide [1]. Within the last decades mental disorders among children and adolescents have been recognized as a global public health concern as they have shown to be associated with suffering, functional impairment, exposure to stigma and discrimination, as well as enhanced risk of premature death [2, 3]. According to global epidemiological data, 13–23% of children and youth suffer from a mental disorder [4, 5]. Early prevention of mental disorders can reduce morbidity risk and may avoid the need for more expensive interventions [6, 7].

Although mental disorders begin already early in life, they are often not detected until adulthood [8–10]. Kessler et al. [10] presented an overview of the age of onset distributions of mental disorders, focusing on the World Health Organization’s World Mental Health surveys. They showed that approximately half of all lifetime mental disorders begin during adolescence. Research on preschool mental health indicates that MHPs show substantial stability and are strongly predictive of mental disorders in adolescence [8, 11, 12]. Within a population-based cohort Basten et al. [13] examined the stability of internalizing and externalizing problems from children aged 1.5–6 years. Their results indicate an overall stability of MHPs through the preschool years.

In addition, Basten et al. [13] showed that a heterotypic continuity of symptom patterns is very common when children get older. As the presentation of problem behaviour changes across the preschool period, the distinction between common, transient problems and those that may be precursors to clinical disorders becomes challenging [14]. Therefore it is not enough to assess only children’s MHPs as indicators for subsequent mental disorders. Mental health problems in preschool-aged children are often affected by risk and protective factors [15, 16]. In order to reduce the associated burden of MHPs and to prevent the development of mental disorders, it is of special interest to identify factors that may serve as indicators of possible future mental problems. Previous research identified numerous personal, biological, and social factors that are positively associated with MHPs in preschool-aged children and increase the likelihood of negative mental health outcomes [17, 18]. These factors are defined as *risk factors* in the present study.

### Parental mental health problems

Findings from cross-sectional as well as longitudinal studies show that one of the most important risk factors for the development of maladaptive emotional and behavioural outcomes in children is MHPs in a parent [19–24]. In a meta-analysis of 193 studies Goodman et al. [25] revealed that maternal depression for example was moderately associated with higher levels of internalizing, externalizing, and general psychopathology in children. Within a prospective longitudinal study Laucht et al. [26] found that preschool-aged children of mentally ill parents displayed higher scores for behaviour problems as compared to children of healthy parents. Within a community sample Hanington et al. [27] reported an adverse effect of parental depression on children’s difficult temperamental characteristics. Goodman et al. [25] therefore concluded that more studies are needed that take into account several child, family and social variables next to parental MHPs, as the adverse influence of parental MHPs on children’s mental health may be increased by other factors.

### Child temperament

Looking at early childhood precursors of subsequent MHPs, children’s difficult temperamental characteristics have been posited as a moderate and consistent risk factor in cross-sectional and longitudinal studies [28, 29]. Temperament is described as relatively persistent over time and is able to predict emotional and behavioural responses of children based on inherent differences in reactivity and self-regulation [30]. Children’s temperament has been shown to be an important factor in understanding the development of MHPs. Coté et al. [31] investigated the onset and developmental course as well as risk factors of depression and anxiety symptoms in a representative population sample of preschool-aged children. Results from annual maternal ratings from infancy to school entry showed that difficult temperament and maternal history of major depressive disorder predicted greater depressive and anxiety symptoms during early childhood. Similar results were reported by Dougherty et al. [32] who examined the association between temperament at age 3 and maternal reports of children’s depressive symptoms at ages 7 and 10. Lower positive and higher negative emotionality are two central traits of a difficult temperament. Children with this pattern at age 3 show the greatest increase in depressive symptoms at age 10. These findings indicate that a difficult temperament in preschool-aged children is a risk factor for emotional disturbance in later childhood. Although temperament is discussed to be biological in origin its effects on children’s mental health may be influenced by environmental conditions [33]. As temperament seems to interact with environmental conditions and may serve as an important risk factor for the development of mental disorders more research attention on temperament in the early developmental period should be paid.

### Parental socioeconomic status

Past research suggests that a low parental SES has a negative impact on children’s mental health and children from low-SES families suffer more often from mental disorders [17, 34–36]. The association between low SES and both social and emotional development are less consistent than the association of SES with children’s cognitive development [34]. In an Australian cross-sectional study Steele et al. [36] identified an association between indicators of social disadvantage and emotional and behavioural difficulties as measured with the SDQ in a general population sample of children aged 4–7 years. The evidence for an association between low SES and MHPs in preschool-aged children is variable. This can be partly attributed to various instruments used to measure MHPs and the different indicators used to establish SES [34–36]. The association between low SES and MHPs becomes strongly evident with increasing age [34].

### Protective factors

Research on protective factors for mental health has shown that the social environment of a child, including their family, peer, school, and neighbourhood contexts [37] is associated with the extent of their developmental resilience. Regarding family and peer context, earlier research found social support to be a protective factor that decreased the risk of children’s development of behaviour problems [38–40]. Furthermore, parental self-perceived competence, which includes perceived self-efficacy as a parent and satisfaction derived from parenting, was shown to be related to child and family functioning [41–43]. Parents with high parental competence are more likely to apply effective parenting strategies, which in turn improve children’s outcomes related to academic and social-psychological domains [43].

Until today studies that specifically focus on preschool-aged children are still not very common, especially in comparison to research on older children and adolescents [14, 15]. As the development of mental disorders is influenced by the caregiving environment, child characteristics and social factors, it is important to gather more information on risk and protective factors in these domains [44]. This information is also important to inform the design of preventive interventions.

In Germany, there have been few studies on correlates of MHPs in preschool-aged children conducted in the general population. Therefore the first goal of the current study was to investigate possible correlates of MHPs in a population-based sample of preschool aged-children in Germany. For this purpose, logistic regression analyses of possible risk and protective factors related to children’s MHPs were performed. Based on findings from previous research, we expected to find that parental MHPs and children’s difficult temperament would be positively related to MHPs in preschoolers. However, because of little evidence regarding the association of low SES and MHPs in very young children, we did not expect to find a statistical significant association between these two factors. Regarding the protective function of parental social support and self-perceived competence, we hypothesized a negative association with children’s MHPs. The second goal of the study was to assess the relative importance of the identified risk and protective factors in the explained variation of MHPs in preschoolers using a hierarchical logistic regression analysis.

## Methods

### Study design and setting

In the BELLA preschool study, families with children aged 3–6 years were randomly recruited from the mental health module of the German Health Interview and Examination Survey for Children and Adolescents (KiGGS) in the third round of data collection between 2005 and 2006 [45]. To gather a nationwide representative sample of children aged 0–17 years, the sample selection for the KiGGS study was performed in two steps. In the first step, 167 sample units were drawn from Germany communities stratified according to their grade of urbanization and geographic distribution. In the second step, the same number of addresses was randomly drawn for each birth cohort within selected sample units. For more details on the sampling methodology, please see Kurth et al. [45]. The BELLA preschool study took place in 33 out of the 167 sample units of the KiGGS study that were distributed equally across Germany and cover all sizes of municipalities in Germany. In each sample unit, 24 families were randomly selected and asked to participate in the BELLA preschool study. Of the 792 families, 450 (49.4%) provided informed consent, of which 391 (87%) gave information on the mental health status of their preschool-aged children. The data were collected with paper–pencil questionnaires, which were sent to all of the families after receiving their informed consent.

### Measures

All measures were completed by a single caregiver for each preschool-aged child. The data was collected using self-completed questionnaires, and the decision on which caregiver’s data was included was that of the caregiver who opted to provide this information.

#### Children’s mental health

To screen for overall MHPs of preschoolers, the German version of the SDQ [46] was completed by parents. The items of the SDQ refer to their rating of the child’s behaviour over past 6 months. The questionnaire contains 25 items that need to be rated on a three-point Likert scale as ‘0 = not true,’ ‘1 = somewhat true,’ or ‘2 = certainly true’. The 25 items are divided between five subscales. The sum of four of the five subscales (range 0–40) adds up to the total difficulties score including emotional symptoms, conduct problems, hyperactivity/inattention, and peer problems. The fifth subscale assessing prosocial behaviour is not incorporated in the total difficulties score. The total difficulties score can be categorized into ‘normal,’ ‘borderline’ and ‘abnormal’ scores. The three categories were classified according to available German normative data [46]. Scores of 13–15 were classified as ‘borderline’ and scores of 16–40 as ‘abnormal’ SDQ scores. The ‘borderline’ and ‘abnormal’ SDQ total difficulties scores predict that mental health disorders are possible or probable [47]. Because the aim of the present study was to assess the associations of risk and protective factors with MHPs, children with ‘borderline’ and ‘abnormal’ total difficulties scores were grouped into one category, ‘borderline or abnormal’. Children with a ‘borderline or abnormal’ SDQ total difficulties score were considered to be at risk for child mental health disorders. The same procedure was applied by Holling et al. [48] in the analysis of the KiGGS study. The German translation of the SDQ has been shown to have a good internal consistency (Cronbach’s alpha 0.83) for the total difficulties score in a clinical sample of 543 children and adolescents [47, 49]. Furthermore, the questionnaire discriminates well between children with and without MHPs [50, 51]. In the BELLA preschool study, the internal consistency (Cronbach’s alpha) was 0.77 for the total difficulties score.

#### Assessment of risk factors

To try to identify clear risk factors for child MHPs, parental mental health, children’s difficult temperament as well as low parental SES were assessed.

#### Parental MHPs

Parental MHPs were assessed with the 9-item version of the Symptom-Check List (SCL-S-9) [52]. The SCL-S-9 is a one-dimensional short version of the SCL-90-R developed by Derogatis et al. [53]. This is a brief screening self-report instrument that indicates a number of psychopathologic symptoms, including somatization, obsessive–compulsive, interpersonal sensitivity, depression, anxiety, hostility, phobic anxiety, paranoid ideation, and psychoticism. Parents answered questions concerning their symptom severity during the last 7 days on a 5-point Likert-scale ranging from ‘0 = none at all’ to ‘4 = very severe’. The mean of all of the item responses represents a global severity index, with higher values indicating more severe MHPs. Two groups were established indicating presence and absence of caregiver MHP risk, with the children being categorized into two groups: with and without caregiver MHPs. The cut-off score was chosen according to Klaghofer and Braehler [52] using the mean plus two standard deviations to indicate the presence of significant caregiver MHPs. The SCL-S-9 showed good reliability and significant correlations with other mental health measures [52, 54]. In the present study, the internal consistency (Cronbach’s alpha) was 0.84.

#### Children’s temperament

The temperament of the child was assessed with the short version of the Temperament Assessment Battery for Children (TABC) [55]. The original form of the questionnaire was developed by Martin in 1988 [56] to measure the temperamental characteristics of children aged 3–7 years. For reasons of practicability, a short version of the TABC by Newman et al. [55] was applied in the BELLA preschool study. The short 15-item version is based on the longer 48-item battery [56]. The short form assesses the five following temperament dimensions: activity level, adaptability/agreeableness, negative emotionality, persistence, and social inhibition. Parents were asked to assess the current behaviour of their children on a five-point Likert scale ranging from ‘0 = never’ to ‘4 = very often’. A higher sum-score over all items indicates that the child has a more difficult temperament. To differentiate between children with an easy temperament and a difficult temperament, the sum-score (ranging from 0 to 60) was categorized. A cut-off score was chosen for the total scale at the 90th percentile of the sample distribution. The children in the 10% of the distribution showing the highest scores on the scale were defined as children with a difficult temperament. Temperamental difficultness was characterized as the combination of extreme activity, low agreeableness and persistence, high negative emotionality, and social inhibition. The short form of the TABC showed satisfactory internal consistency, independence of the temperament dimensions, and satisfactory validity [55]. The internal consistency for the short version of the TABC used in the BELLA preschool study, as measured by Cronbach’s alpha, was 0.72.

#### Parental socioeconomic status

The parental SES was assessed according to Winkler and Stolzenberg [57]. This approach determines SES from parental education, profession and income. The total sum-score of the index ranges from 3 to 21 and was divided into three categories of low (scores from 3 to 8), middle (scores from 9 to 14) and high SES (scores from 15 to 21) [58].

#### Assessment of protective factors

Within the BELLA preschool study,  two protective factors were assessed: parental social support and parental competence.

#### Social support

To assess parental perception of perceived social support, the surveyed parents were asked how much social support they had received during the child’s first year of life [“If you think of the first year of your child’s life, how supported did you feel by others (exp.: spouse, family, friends)?”]. Answers were coded on a 3-point Likert-scale ranging from ‘0 = no support’ to ‘2 = strong support’.

#### Parental competence

The parental competence was assessed with the German version [59] of the Parenting Sense of Competence Scale (PSOC) [60]. The PSOC is the most commonly used questionnaire for measuring general parental self-efficacy [43] and assesses parental competence with the use of two subscales: perceived self-efficacy in the parenting role and satisfaction with parenting. Parents were asked to assess their level of agreement with 16 items on a 6-point Likert-scale ranging from ‘1 = strongly disagree’ to ‘6 = strongly agree.’ According to Johnston and Mash [60], the total sum-score over all items was categorized into high competence (74–96), moderate competence (61–73) and low competence (16–60). The reliability of the subscales and overall scale vary from an alpha of 0.70 to 0.79 [59, 60]. In the present study, the internal consistency for the overall scale score, as measured with Cronbach’s alpha, was 0.82.

### Statistical analyses

To ensure the representativeness of the sample regarding gender, age, SES and geographic region, post hoc case weights were calculated based on reference data from the German Federal Office of Statistics (31 Dec 2012). For more details on the weighting method, please see Wlodarczyk et al. [61]. All analyses were conducted with the weighted data. To test the robustness of the findings, we performed an additional sensitivity analysis in which we repeated all calculations using the unweighted data.

To determine the collinearity between all of the predictor variables, Phi coefficient and Cramer’s V were calculated. The associations between the predictor variables were classified as weak to moderate and ranged from 0.02 to 0.37.

The frequencies of predictor variables (temperament of the child, parental psychopathology, SES and competence, social support in the first year of the child’s life) were calculated with 95% confidence intervals (CI). To examine to what extent the included predictors can be regarded as risk or protective factors for MHPs in preschool-aged children, the associations of predictor and sociodemographic variables with MHPs were examined using logistic regression analyses. First, bivariate logistic regression analyses were conducted to assess the association of each variable with MHPs (dependent variable). Overall, eight variables were evaluated that may impact MHPs in preschoolers. These included gender and age of the child, geographic area, parental SES, temperament of the child, parental psychopathology and competence, and parental social support in the first year of the child’s life. In the second step, three hierarchically structured multivariate logistic regression models including all of the sociodemographic and predictor variables were examined:

1. A model that considered only the sociodemographic variables;
2. A model that included potential risk factors (parental MHPs, child’s difficult temperament, low parental SES) in the analysis,
3. And a model that added potential protective factors (parental social support and competence) in the analysis.

For all of the associations, odds ratios (ORs) with 95% CI were calculated.

The percentage of missing values per child and item was maximal 6.1% in the present study. Maximum likelihood estimation (EM algorithm) was used to estimate missing values [62, 63]. The EM algorithm is used to estimate maximum likelihood parameters in probabilistic models with incomplete data. The EM algorithm involved two steps: the expectation step (E-step) and the maximization step (M-step). In the E-step, missing values were estimated based on the observed data and current model parameters. In the M-step, current model parameters are re-estimated using the maximum likelihood estimation procedure based on the completed data. Both steps were repeated until there was convergence [62, 63].

In the present study, multiple statistical tests were performed on the same sample of data. To protect against an increased risk of the type I error, the Bonferroni adjustment was applied. In the Bonferroni-adjusted analyses, the nominal significance level of 0.05 was divided by the number of predictors in the model. Because the number of predictor variables in the current model was eight, we considered the findings to be statistically significant at p < 0.006. All analyses were conducted using SPSS version 18 for Windows [64].

## Results

### Participants

In total, 51.7% of the preschoolers were female, 49.6% were 3–4 years and 50.4% were 5–6 years old. Most children (80.8%) lived in western Germany. The sociodemographic data of the sample including a comparison with the data from the Federal Office of Statistics are presented in the previous published paper by Wlodarczyk et al. [61].

In 81.2% of the cases, the questionnaire was answered by the biological mother, in 14.8% by the biological mother together with the biological father, in 2.8% by the biological father, and in 1.1% by another caregiver (a step- or grandparent or an adoptive or a fostering parent).

### Frequencies of risk and protective factors

Based on the caregivers’ assessment of the SCL-S-9 5.9% (95% CI 3.6–8.2) of the children had parents suffering from MHPs. In 8.6% (95% CI 5.8–11.4) of the cases parents judged their children to show a difficult temperament and approximately one quarter of the parents could be classified as low SES (26.8%; 95% CI 22.4–31.2). A total of 70.0% (95% CI 65.5–74.6) of the parents perceived that they received strong social support during the child’s first year of life. About 17.0% (95% CI 13.3–20.7) assessed their social support as moderate and 13.0% (95% CI 9.7–16.3) as not existend during that time. Most of the parents judged their parental competence as high (46.4%; 95% CI 41.5–51.3), 44.6% (95% CI 39.7–49.5) as moderate and 9.1% (95% CI 6.3–11.9) as low.

### Effect of predictors on MHPs in preschool-aged children

In this study, 18.2% (95% CI 14.4–22.0) of the preschool-aged children were categorized as being ‘borderline or abnormal’ based on their total difficulties score on the SDQ and were thus defined as being at risk for mental health disorders.

Bivariate logistic regression analyses revealed that MHPs in preschoolers were statistically significantly associated with parental MHPs, children’s temperament, parental SES and parental competence (see Table 1). Increased likelihood of MHPs in preschoolers was associated with parental MHPs (OR 3.68, 95% CI 1.54–8.80). A child’s difficult temperament (OR 5.30, 95% CI 2.54–11.06) was linked to a statistically significant increased likelihood that the child would show MHPs. Parental low SES was associated with a 2.47-fold (95% CI 1.28–4.78) increase in the likelihood that preschoolers would show MHPs.

**Table 1:** Bivariate logistic regression analyses of risk and protective factors for MHPs in preschoolers (*N* = 391), weighted data

	% (n)	Bivariate OR	(95% CI)	p^a^
Sociodemographic characteristics				
Gender				
Male	51.7 (202)	Ref.		
Female	48.3 (189)	0.64	(0.38–1.08)	0.09
Age (years)				
3–4	49.6 (194)	Ref.		
5–6	50.4 (197)	0.83	(0.49–1.38)	0.47
Geographical region				
East	19.2 (75)	Ref.		
West	80.8 (316)	1.45	(0.71–2.94)	0.30
Risk factors				
Parental mental health				
Not impaired	94.1 (368)	Ref.		
Impaired	5.9 (23)	3.68	(1.54–8.80)	*0.003*
Child’s temperament				
Easy	91.4 (357)	Ref.		
Difficult	8.6 (34)	5.30	(2.54–11.06)	*0.000*
Parental SES				
High	30.1 (118)	Ref.		
Middle	43.1 (169)	0.90	(0.46–1.77)	0.76
Low	26.8 (104)	2.47	(1.28–4.78)	0.007
Protective factors				
Parental social support				
Low	13.0 (51)	Ref.		
Moderate	16.9 (66)	0.77	(0.33–1.80)	0.55
High	70.0 (274)	0.50	(0.25–1.01)	0.05
Parental competence				
Low	46.4 (181)	Ref.		
Moderate	44.6 (175)	0.30	(0.14–0.64)	*0.002*
High	9.1 (35)	0.21	(0.09–0.46)	*0.000*

Italics statistically significant

*MHPs* mental health problems, *OR* odds ratio, *95% CI* 95% confidence interval, *Ref*. reference category, *SES* socioeconomic status

^a^p value determined using bivariate logistic regression analyses

Caregivers with moderate (OR 0.30, 95% CI 0.14–0.64) or high (OR 0.21, 95% CI 0.09–0.46) perceived parenting competence were significantly less likely to have children with MHPs. No significant effects on children’s MHPs were found for child’s gender and age, geographical region and parental social support during the first year of child’s life.

To explore the functional relationships between the sociodemographic and predictor variables and preschoolers’ MHPs, a series of hierarchical logistic regression analyses were conducted (see Table 2). In testing the first model, the associations between all sociodemographic variables and preschoolers’ MHPs were examined (see Table 2). Again, sociodemographic variables were not statistically significant associated with MHPs.

**Table 2:** Hierarchical multivariate logistic regression analysis of risk and protective factors for MHPs in preschoolers (*N* = 391), weighted data

Predictors	Model 1			Model 2			Model 3		
	OR	95% (CI)	p^a^	OR	95% (CI)	p	OR	95% (CI)	p
Sociodemographic variables									
Gender									
Male	Ref.			Ref.			Ref.		
Female	0.64	(0.38–1.07)	0.089	0.67	(0.39–1.17)	0.159	0.65	(0.37–1.13)	0.122
Age (years)									
3–4	Ref.			Ref.			Ref.		
5–6	0.84	(0.50–1.40)	0.50	0.92	(0.53–1.59)	0.757	0.89	(0.51–1.56)	0.692
Geographical region									
East	Ref.			Ref.			Ref.		
West	1.47	(0.72–2.98)	0.29	1.83	(0.84–3.95)	0.126	1.94	(0.88–4.31)	0.103
Risk factors									
Parental mental health									
Not impaired	–	–	–	Ref.			Ref.		
Impaired	–	–	–	2.76	(1.05–7.29)	0.040	2.06	(0.69–6.15)	0.193
Child’s temperament									
Easy	–	–	–	Ref.			Ref.		
Difficult	–	–	–	4.83	(2.20–10.61)	*0.000*	3.85	(1.67–8.85)	*0.002*
Parental SES									
High	–	–	–	Ref.			Ref.		
Middle	–	–	–	0.84	(0.42–1.71)	0.636	0.84	(0.41–1.72)	0.64
Low	–	–	–	2.18	(1.09–4.36)	0.028	2.12	(1.04–4.31)	0.039
Protective factors									
Parental social support									
Low	–	–	–	–	–	–	Ref.		
Moderate	–	–	–	–	–	–	1.08	(0.42–2.82)	0.868
High	–	–	–	–	–	–	0.98	(0.41–2.32)	0.961
Parental competence									
Low	–	–	–	–	–	–	Ref.		
Moderate	–	–	–	–	–	–	0.42	(0.17–1.00)	0.051
High	–	–	–	–	–	–	0.36	(0.14–0.90)	0.029
Model accuracy									
Nagelkerkes R^2^	0.02			0.15			0.17		
Hosmer–Lemeshow test	χ^2^ 1.09, *p* = 0.96			χ^2^ 4.71, *p* = 0.79			χ^2^ 7.73, *p* = 0.46		

Italics statistically significant

*MHPs* mental health problems, *OR* odds ratio, *95% CI* 95% confidence interval, *Ref*. reference category, *SES* socioeconomic status

^a^p value determined using bivariate logistic regression analyses

In the second model, children’s difficult temperament remained significantly related to MHPs when controlling for sociodemographic variables, and parental mental health in addition to SES. A difficult temperament (OR 4.83, 95% CI 2.20–10.61) was related to an increased likelihood of showing MHPs. Compared to the bivariate logistic regression analyses, parental MHPs no longer reached statistical significance. The predictive power of the second model was improved with Nagelkerke’s *R* ^2^ of 0.16.

In the full model (model 3), possible protective factors (parental social support and competence) were also included in the logistic regression. Again, children’s difficult temperament (OR 3.85, 95% CI 1.67–8.85) remained the only significant predictor of children’s MHPs. In the full model, adding parental social support and competence to the regression no longer improved the fit of the model to the data as the model accuracy showed only marginal improvement compared to the second model (*R* ^2^ 0.17).

### Sensitivity analyses

For results of all calculations repeatedly conducted with the unweighted data, see Additional file 1: Tables S1, S2. The results of the bivariate logistic regression analyses conducted with the unweighted data showed similar results to the bivariate analyses conducted with the weighted data. Parental MHPs (OR 7.85, 95% CI 3.47–17.75) and children’s difficult temperament (OR 7.71, 95% CI 3.84–15.47) were, as in the analysis with the weighted data, associated with an increased likelihood of MHPs. Moderate (OR 0.28, 95% CI 0.13–0.57) and high (OR 0.15, 95% CI 0.07–0.32) parental competence were related to a significant decrease in the likelihood of MHPs in preschoolers. Except for the results of the first model of the multivariate analyses, different results existed between the weighted and unweighted data. In particular, in the second model of the multivariate analysis, next to children’s difficult temperament (OR 5.61, 95% CI 2.60–12.10), parental MHPs (OR 6.58, 95% CI 2.57–16.84) remained significantly related to an increased likelihood of children’s MHPs. In the full model (model 3), parental MHPs (OR 5.29, 95% CI 1.76–15.94) and children’s difficult temperament (OR 4.53, 95% CI 2.03–10.09) were again associated with an increase in the likelihood of MHPs controlled for sociodemographic and protective factors. The full model of the unweighted data showed better model accuracy (R^2^ 0.25, χ^2^ 7.98, *p* = 0.44) compared to the weighted data (R^2^ 0.17, χ^2^ 7.73, *p* = 0.46).

## Discussion

In the bivariate analysis preschool-aged children with a difficult temperament and whose parents showed MHPs faced a higher risk and children of parents with middle or high parental competence faced a lower risk for the development of MHPs. Some factors such as parental SES and social support, previously discussed as associated factors [38–40], did not show significant associations with MHPs in the present study. Considering all of the sociodemographic and risk factors at once in a multivariate model showed that only children’s difficult temperament remained significantly associated with an increased risk of MHPs. Including protective factors in the next step of the regression analysis did not change this association. These results indicate that children’s temperament best predicts MHPs in preschoolers when controlling for sociodemographic variables, parental MHPs and SES as well as social support and parental competence.

Results of the present study underline the importance of preschoolers’ difficult temperament as a risk factor for mental health in a representative sample of the general population. Comparable associations could be shown by previous studies which investigated the influence of difficult temperament in preschool-aged children with high levels of internalizing and externalizing problems [29, 65]. Findings of the present study suggest that an early identification of a difficult temperament can serve as an indicator of subsequent MHPs in preschool-aged children. The importance of the identification of a difficult temperament in early childhood was demonstrated in a British longitudinal study conducted by Bould et al. [66]. Their results underlined that high levels of negative emotionality in children at age 6 showed to be good predictors of depressive disorders at age 18. Previous literature has tried to explain the association between temperament and MHPs with the conceptual overlap between measures [67]. This measurement confounding was examined in a study by Lemery et al. [68] who showed that the removing of confounded items from measures did not affect the association between temperament and MHPs. In the present study results show that there is no perfect association between both constructs. Therefore relation between temperament and MHPs cannot be fully explained by a conceptual overlap. According to the model accuracy of multivariate regression analyses in the present study, the effects of children’s temperament on MHPs have to be considered as modest. However, comparably weak effects of temperament on psychopathology have been found in previous studies [69]. This modest amount of explained variation suggests that additional factors are likely to influence children’s MHPs. Although it is argued that temperament is to some degree biologically based, recent literature indicates that children with difficult temperaments were more susceptible for environmental influences than children with an easy temperament [65]. Mesman et al. [65] revealed that toddlers with a difficult temperament showed a higher decrease in externalizing problems when experiencing sensitive parenting as toddlers with an easy temperament. The development of MHPs in children with a difficult temperament seem to be influenced by reactions and responses of significant others to the children’s behaviour [67]. Children with a difficult temperament are an important risk group as they are more vulnerable to a poor outcome and should therefore be considered in preventive interventions [67]. More knowledge about the influence of child’s difficult temperament on the child’s family and social environment may be important in the development of preventive interventions. The prevention program INSIGHTS into Children’s Temperament by McClowry et al. [70] teaches for example parents and teachers how to apply temperament-based child management strategies to reduce behaviour problems and enhance children’s empathy and problem-solving skills in school-aged children.

The association of parental psychopathology with children’s MHPs was shown in the bivariate analysis and is in line with the results of previous studies [39, 71]. Contrary to our assumptions, parental MHPs did not predict children’s MHPs in the full model of the logistic regression analysis. However, the calculations conducted with the unweighted data showed different results in the multivariate analyses. Here, parental MHPs remained significantly associated with children’s MHPs in the full model. As the aim of the study was to present results for a representative sample of German preschool-aged children, post hoc case weights were applied in order to adjust for a disproportionate representation of the population. The weighting resulted in different estimates in certain categories with a smaller relative quantity of parents with MHPs in the weighted sample. This result does not suggest that parental MHPs is not important for the development of children’ MHPs. Previous research on the association of parental and children’ MHPs showed that the characteristics of parental mental disorder play an important role in the explanation of this association. In particular, parents with severe, chronic mental disorders who subjectively experience impairments pose a higher risk for the development of children’s MHPs [72, 73]. As the present study was conducted in the general population the association of parental MHPs with children’s MHPs may be weaker compared to other studies conducted in clinical samples. Furthermore, the decision of which caregiver’s data was included in the study was solely made by the caregiver who opted to provide this information. It therefore possible that caregivers who were willing to participate were less likely to be depressed, anxious or paranoid.

As found in the previous publication based on the BELLA preschool study dataset [61], the results of bivariate and multivariate analyses in the present study were at variance with studies from the previous literature on the association between parental SES and children’s MHPs [17, 34–36]. The association of parental SES and children’s MHPs were considered not to be statistical significant after the Bonferroni adjustment was applied. However, the confidence intervals show a strong association at 5.30 (2.54–11.06) between both factors. Although the p value did not reach statistical significance to the adjusted <0.006, there still appears to be a relationship between low parental SES and MHPs due to the odds ratio and the confidence intervals.

It must be asked to what extent SES can be used as a measure of socioeconomic differences in the present study. Flouri [33] underlined two problematic aspects of using SES to assess socioeconomic differences by questioning if SES is a sufficiently sensitive measure and if it can be used as an indicator of a latent social dimension.

The bivariate analysis further indicated that high parental competence reduces the risk of children’s MHPs. This finding is consistent with previous studies on parental competence showing that perceived parental competence was associated with children’s developmental outcomes [43, 74, 75]. Knoche et al. [74] demonstrated that the interaction of depression and maternal sense of competence significantly predicted children’s cognitive development. Despite high levels of depression, mothers with high parental competence were able to foster positive outcomes in their infants [74]. However, in the multivariate regression, the significant association of parental competence and children’s MHPs did not remain significant. This result showed that caregiver perception of self-competence did not predict children’s MHPs in the present study when other variables where controlled.

This study had several strengths: We investigated a representative sample of the general population of German preschoolers with regard to the distribution of gender, age, parental SES, and geographical region. We analysed the potential influences of children’s temperament, parental MHPs and further psychosocial factors on children’s MHPs in an age group for which so far in Germany there have been few studies. Despite the strengths, several limitations have to be addressed. No non-responder analysis could be conducted in the present study, because the data of parents who did not respond were not available. Furthermore, the sample cannot be considered as representative regarding the distribution of migration background and community area. Because the BELLA preschool study has been a cross-sectional study, it is difficult to draw conclusions about whether the significant factors play a role in the onset of MHPs or whether they are simply correlates or consequences of MHPs. However, the identification of risk factors in early childhood can serve as indicators of possible MHPs. More information on causal influence of risk and protective factors can be derived from studies utilizing longitudinal designs. Furthermore, the sample size of the present study has to be considered as moderate, resulting in relatively small numbers of events per predictor variable in some cases. Several outcomes were close to reaching statistical significance which may indicate that the study was not adequately powered. Therefore, the results should be interpreted with caution. Furthermore, the present study included only a limited number of risk and protective factors. It must be considered that these factors may be influenced or mediated by additional factors that were not assessed as for example parental discipline, child physical health, or family grief and illness events [15].

Additionally, the present study relied solely on parental reporting. Information on children’s temperament were relied upon the same caregiver who also assessed his/her mental health, social support and competence. As the outcomes were not validated by an independent assessment the extent to which parent reports are valid and reliable should be considered. Stifter et al. [76] compared judgments of children’s temperament behaviours made by parents and external observers and found that parent and observer ratings diverged in assessing negative aspects of children’s temperament. Therefore, it is possible that the use of parent-based measurements only resulted in substantial bias.

Stronger evidence from studies with larger sample sizes that use multiple informants is necessary. Furthermore, the validity of some measurements has to be critically examined. The present study applied screening instruments to assess parental and children’s mental health. These instruments only indicate MHPs and do not imply clinical diagnoses. Moreover, the assessment of parental social support was based on a one-item questionnaire. The application of better validated, more reliable measures and the inclusion of independent assessment of infant temperament and behaviour would have yielded different results. Our conceptualization of children’s temperament was relatively rough and did not differentiate between specific temperament dimensions.

## Conclusions

The aim of the present study was to investigate possible correlates of MHPs in preschool-aged children within a German community sample. Using logistic regression analyses possible risk and protective factors of MHPs were identified and their relative importance was assessed using a hierarchical logistic regression analysis. Contrary to expected results, preschoolers’ difficult temperament showed to be the only statistically significant factor positively associated with MHPs controlled for parental SES, MHPs, social support, and competence as well as sociodemographic variables. These results highlighted the meaning of temperament as a risk factor for mental health in preschoolers and underlined the importance of strengthening the mental health of children with a difficult temperament in prevention and intervention programs. Results of the present cross-sectional study need to be replicated in further prospective studies in order to examine the influence of difficult temperament over time and its interaction with environmental factors.

## Additional file

**Additional file 1: Table S1.** Bivariate logistic regression analyses of risk and protective factors for MHP in preschoolers (*N* = 391), unweighted data. **Table S2.** Hierarchical multivariate logistic regression analysis of risk and protective factors for MHP in preschoolers (*N* = 391), unweighted data.

## Abbreviations

MHPs: mental health problems
SDQ: Strength and Difficulties Questionnaire
SES: socioeconomic status
KiGGS: German Health Interview and Examination Survey for Children and Adolescents
SCL-S-9: Symptom-Check List 9-item Short version
TABC: Temperament Assessment Battery for Children
PSOC: Parenting Sense of Competence Scale
OR: odds ratio
CI: confidence intervals
EM: expectation–maximization
E-step: expectation step
M-step: maximization step
Ref: reference category
