# Supplementary material for: Risk and protective factors for mental health problems in preschool-aged children: cross-sectional results of the BELLA preschool study
Source: Child Adolesc Psychiatry Ment Health. 2017 Mar 8;11:12. doi: 10.1186/s13034-017-0149-4 (PMC5341413; doi:10.1186/s13034-017-0149-4)
Supplement: Supplementary file 1 — Additional file 1: Table S1. Bivariate logistic regression analyses of risk and protective factors for MHP in preschoolers (N = 391), unweighted data. Table S2. Hierarchical multivariate logistic regression analysis of risk and protective factors for MHP in preschoolers (N = 391), unweighted data. [file 13034_2017_149_MOESM1_ESM.docx]

**Supporting Information**

| **Additional Table S1. Bivariate Logistic Regression Analyses of Risk and Protective Factors for MHP ^a^ in Preschoolers (*N* = 391), Unweighted Data** | | | | |
| --- | --- | --- | --- | --- |
|  | | **Bivariate OR ^b^** | **(95 % CI) ^c^** | **P ^d^** |
| **Sociodemographic Characteristics** | |  | | |
| Gender | Male | Ref ^e^ |  |  |
|  | Female | 0.55 | (0.32-0.85) | 0.033 |
| Age (years) | 3-4 | Ref. |  |  |
|  | 5-6 | 0.94 | (0.54-1.59) | 0.804 |
| Geographical Region | East | Ref. |  |  |
|  | West | 1.04 | (0.57-1.91) | 0.889 |
| **Risk Factors** | |  |  |  |
| Parental Mental Health | Not impaired | Ref. |  |  |
|  | Impaired | 7.85 | (3.47-17.75) | **0.000** |
| Children’s Temperament | Easy | Ref. |  |  |
|  | Difficult | 7.71 | (3.84-15.47) | **0.000** |
| Parental SES ^f^ | High | Ref. |  |  |
|  | Middle | 0.95 | (0.46-1.98) | 0.898 |
|  | Low | 2.60 | (1.27-5.33) | 0.009 |
| **Protective factors** | |  |  |  |
| Parental Social Support | Low | Ref. |  |  |
|  | Moderate | 0.70 | (0.31-1.63) | 0.411 |
|  | High | 0.41 | (0.21-0.82) | 0.012 |
| Parental Competence | Low | Ref. |  |  |
|  | Moderate | 0.28 | (0.13-0.57) | **0.001** |
|  | High | 0.15 | (0.07-0.32) | **0.000** |

^a^ *MHP*, mental health problem; ^b^ *OR, odds ratio;* ^c^ *95 % CI*, 95% confidence interval*;* ^d^ P value determined using bivariate logistic regression analyses; ^e^ *Ref*., reference category; ^f^ *SES*, socioeconomic status; **bold** = statistically significant

| **Additional Table S2. Hierarchical Multivariate Logistic Regression Analysis of Risk and Protective factors for MHP ^a^ in Preschoolers (*N* = 391), Unweighted Data.** | | | | | | | | | | | | |
| --- | --- | --- | --- | --- | --- | --- | --- | --- | --- | --- | --- | --- |
|  | | | **Model 1** | | | | **Model 2** | | | **Model 3** | | |
| **Predictors** | | | **OR ^b^** | | **95% (CI) ^c^** | **P ^d^** | **OR** | **95 % (CI)** | **P** | **OR** | **95 % (CI)** | **P** |
| **Sociodemographic variables** | | | | | | | | | | | | |
| Gender | Male | | Ref. ^e^ | |  |  | Ref. |  |  | Ref. |  |  |
|  | Female | | 0.55 | | (0.32-0.95) | 0.033 | 0.65 | (0.36-1.19) | 0.161 | 0.63 | (0.34-1.15) | 0.131 |
| Age (years) | 3-4 | | Ref. | |  |  | Ref. |  |  | Ref. |  |  |
|  | 5-6 | | 0.94 | | (0.55-1.62) | 0.84 | 1.09 | (0.60-1.97) | 0.780 | 1.05 | (0.58-1.92) | 0.869 |
| Geographical | East | | Ref. | |  |  | Ref. |  |  | Ref. |  |  |
| region | West | | 1.07 | | (0.58-1.97) | 0.82 | 1.44 | (0.72-2.86) | 0.304 | 1.44 | (0.71-2.92) | 0.309 |
| **Risk factors** | | | | | | | | | | | | |
| Parental | | Not impaired | | - | - | - | Ref. |  |  | Ref. |  |  |
| Mental Health | | Impaired | | - | - | - | 6.58 | (2.57-16.84) | **0.000** | 5.29 | (1.76-15.94) | **0.003** |
| Child’s | | Easy | | - | - | - | Ref. |  |  | Ref. |  |  |
| temperament | | Difficult | | - | - | - | 5.61 | (2.60-12.10) | **0.000** | 4.53 | (2.03-10.09) | **0.000** |
| Parental SES ^f^ | | High | | - | - | - | Ref. |  |  | Ref. |  |  |
|  | | Middle | | - | - | - | 0.74 | (0.33-1.64) | 0.636 | 0.77 | (0.34-1.75) | 0.54 |
|  | | Low | | - | - | - | 1.91 | (0.87-4.22) | 0.109 | 1.97 | (0.88-4.42) | 0.100 |
| **Protective factors** | | | | | | | | | | | | |
| Parental | | Low | | - | - | - | - | - | - | Ref. |  |  |
| Social | | Moderate | | - | - | - | - | - | - | 1.37 | (0.47-3.98) | 0.563 |
| Support | | High | | - | - | - | - | - | - | 1.28 | (0.49-3.34) | 0.615 |
| Parental | | Low | | - | - | - | - | - | - | Ref. |  |  |
| Competence | | Moderate | | - | - | - | - | - | - | 0.52 | (0.22-1.27) | 0.151 |
|  | | High | | - | - | - | - | - | - | 0.37 | (0.14-0.97) | 0.043 |
| **Model accuracy** | | | |  | | |  | | |  | | |
| Nagelkerkes R² | | | | 0.02 | | | 0.23 | | | 0.25 | | |
| Hosmer-Lemeshow test | | | | χ² 2.76, *p* = 0.74 | | | χ² 3.33, *p* = 0.85 | | | χ² 7.98, *p* = 0.44 | | |

^a^ *MHP*, mental health problem; ^b^ *OR, odds ratio*; ^c^ *95 % CI*, 95% confidence interval*;* ^d^ P value determined using bivariate logistic regression analyses; ^e^ *Ref*., reference category; ^f^ *SES*, socioeconomic status; **bold** = statistically significant
